# Interconnections between apoptotic, autophagic and necrotic pathways: implications for cancer therapy development

**DOI:** 10.1111/jcmm.12001

**Published:** 2013-01-10

**Authors:** Mayur V Jain, Anna M Paczulla, Thomas Klonisch, Florence N Dimgba, Sahana B Rao, Karin Roberg, Frank Schweizer, Claudia Lengerke, Padideh Davoodpour, Vivek R Palicharla, Subbareddy Maddika, Marek Łos

**Affiliations:** a Department of Clinical & Experimental Medicine, Division of Cell Biology, Integrative Regenerative Med. Center (IGEN), Linköping UniversityLinköping, Sweden; b Department of Hematology & Oncology, University of Tübingen Medical Center IITübingen, Germany; c Department of Human Anatomy and Cell Science, University of ManitobaWinnipeg, MB, Canada; d Division of Otorhinolaryngology, Linköping University HospitalLinköping, Sweden; e Department of Chemistry, University of ManitobaWinnipeg, MB, Canada; f Department of Medical and Health Sciences, Linköping UniversityLinköping, Sweden; g Laboratory of Cell Death & Survival, Centre for DNA Fingerprinting and Diagnostics (CDFD)Hyderabad, India; h BioApplications EnterprisesWinnipeg, MB, Canada

**Keywords:** Bcl2 family, p53, Cancer therapy, Erk, FOXO, PI3-K, Rheb, S6K

## Abstract

The rapid accumulation of knowledge on apoptosis regulation in the 1990s was followed by the development of several experimental anticancer- and anti-ischaemia (stroke or myocardial infarction) drugs. Activation of apoptotic pathways or the removal of cellular apoptotic inhibitors has been suggested to aid cancer therapy and the inhibition of apoptosis was thought to limit ischaemia-induced damage. However, initial clinical studies on apoptosis-modulating drugs led to unexpected results in different clinical conditions and this may have been due to co-effects on non-apoptotic interconnected cell death mechanisms and the ‘yin-yang’ role of autophagy in survival *versus* cell death. In this review, we extend the analysis of cell death beyond apoptosis. Upon introduction of molecular pathways governing autophagy and necrosis (also called necroptosis or programmed necrosis), we focus on the interconnected character of cell death signals and on the shared cell death processes involving mitochondria (*e.g*. mitophagy and mitoptosis) and molecular signals playing prominent roles in multiple pathways (*e.g*. Bcl2-family members and p53). We also briefly highlight stress-induced cell senescence that plays a role not only in organismal ageing but also offers the development of novel anticancer strategies. Finally, we briefly illustrate the interconnected character of cell death forms in clinical settings while discussing irradiation-induced mitotic catastrophe. The signalling pathways are discussed in their relation to cancer biology and treatment approaches.

- Introduction
- Signalling pathways regulating apoptosis
  – Death receptors
  – Caspase family proteases
  – Mitochondria and apoptotic signalling
  – Bcl2 proteins
  – Removal of apoptotic cells
- Autophagy
  – Molecular mechanisms involved in autophagy
  – Regulation of autophagy
  – Autophagy as a survival response to stress
- Cell fate and the interplay between autophagy, apoptosis and necrosis
- Differential effects of autophagy on apoptosis
  – Autophagy as an antagonist of apoptosis
  – Autophagy as a facilitator of apoptosis
  – Autophagy and apoptosis cooperating to induce cell death
- Mitoptosis
- Necrosis
  – Mitochondria and necrosis
  – The role of RIPK1 and RIPK3 in necrosis
- Autophagy–a ‘double-edged sword’ in cancer treatment
- Cellular context matters in irradiation-induced cell death in clinical settings
- Stress-induced cell senescence
- Clinical relevance of cell death modulation

## Introduction

Cell death is of major importance in regulating organismal development, tissue homoeostasis and stress response and interconnects with cell survival and proliferation [1, 2]. During tumour development, uncontrolled cell proliferation is aided by the disablement of cell death responses triggered by specific oncogenes [3–5]. The execution of cell death requires an orchestrated interplay between three important processes: apoptosis, necrosis and autophagy [6]. Cancer, autoimmune diseases, neurodegenerative diseases, and ischaemia-reperfusion damage are the result of the intricate interplay between signalling pathways involving apoptosis, necrosis and autophagy [7]. Various cell death stimuli may trigger some of these pathways simultaneously and/or activate common downstream elements [5, 8–10]. In this review, we outline the characteristics of the different cell death pathways with particular focus on inter-regulatory mechanisms.

## Signalling pathways regulating apoptosis

Apoptosis is an active, specialized form of cell death, and a well orchestrated by a set of hierarchical molecular events. Apoptosis can be triggered either by surface death receptors or through mitochondrial release of cytochrome c, upon conditions, treatments or events causing cellular stress [11]. The common biological denominator of the induction of apoptosis is the activation of caspase family of proteases, which can be detected upon death receptor activation as early as after 10–15 min. [12, 13], whereas the mitochondrial pathways typically requires much more time. Like for other forms of cell death, the morphological definition of apoptosis is the most prevailing and useful. Thus, apoptosis is characterized by loss of cell-cell contact, detachment, cell shrinkage (loss of K+ and water) nuclear condensation, internucleosomal DNA cleavage (CAD-activation), nuclear fragmentation, membrane blebbing and cell-self-fragmentation into apoptotic bodies, that are quickly removed by professional phagocytes and neighbour cells (attracted by membrane-exposure of phosphatidylserine that serves as ‘eat me’ signal). Under optimal conditions, apoptosis may take as little as about 2 hrs (*i.e*. upon CD95-triggering), whereas it often requires several hours if triggered by stress or Tumour Necrosis Factor-α (TNF).

Apoptosis occurs ubiquitously, it is the main feature of tissue development and homoeostasis and represents an active, energy-consuming, suicidal cellular event. Deregulation of apoptosis is of prime importance to the (patho)physiology of multicellular organisms and of major therapeutic interest. Insight into mechanisms of apoptosis is of central importance to drug development as many chemotherapeutic and radiation therapeutic strategies can initiate cell death [14, 15]. Acquired mutations and associated alterations in signalling pathways in tumour cells can cause increased resistance to cell death and are relevant therapeutic targets in tumour therapy. In diseases associated with net cell loss, *i.e*. neurodegenerative disorders or viral infections, the therapeutic goal is to stop or reduce excessive cell death. Of the extensive body of literature existing on apoptosis, we have chosen to select those scientific contributions that identified important interconnections between apoptosis and other cell death pathways. Apoptotic programs may be initiated by a variety of internal and external stimuli. Internal stimuli will mainly initiate apoptosis through the mitochondrial pathway. In contrast, external stimuli engage external pathways *via* activation of death receptors on the cell surface as well as internal pathways that cause cellular stress, *i.e*. irradiation, drugs and chemicals interfering with cell metabolism, viral infection, growth/survival factor deprivation. Clinically, internal pathways like the mitochondrial pathway are more significant as they are commonly triggered by therapeutics and irradiation [15–17]. Classical examples of clinically relevant external, death receptor-triggered death pathways are sepsis (TNF, and Apo1/Fas/CD95 triggered failure of liver and other organs), autoimmunity (failure to remove the surplus of antigen-specific T or B-cells after successful conclusion of immune responses), or negative selection within the immune system.

### Death receptors

Upon interaction with specific ligands (Fas, TNF and TRAIL), cell surface death receptors transmit apoptotic signals [11, 18–21]. The activation of the caspase cascade occurs usually within seconds to minutes of ligand binding [13].

#### TNF/TNF-receptor system as a protoplast of death receptor/ligand family triggering apoptosis

**Fig. 1:**
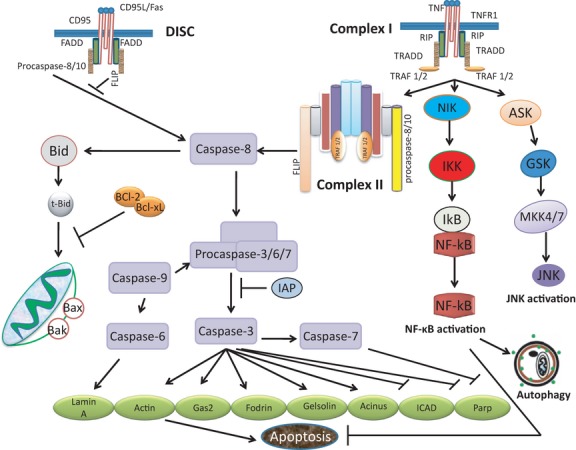
Death-receptor family signalling. Two types of signalling complexes can form at death receptors. Death-inducing signalling complexes known as DISCs are formed at CD95, TRAIL R-1 or TRAIL R-2. These receptors recruit DISCs that have a similar basic composition (FADD, pro-caspases-8). DISC complexes allow caspase-8 activation and transduction of the apoptotic signal. The second group comprises the TNFR1/DR3/DR6 and EDAR receptors which recruit a different set of molecules (please see text for details) that transduces both apoptotic and survival signals.

Tumour necrosis factor-α is a pleiotropic pro-inflammatory cytokine secreted mainly by monocytes/macrophages. TNF affects cell survival and proliferation, insulin resistance, lipid metabolism, coagulation and endothelial function. TNF was first identified in murine serum after injection with Mycobacterium bovis strain Bacillus Calmette-Guerin (BCG) and endotoxin. Serum from these animals was cytotoxic or cytostatic to a number of murine and human transformed cell lines and produced haemorrhagic necrosis and anecdotal tumour regression in experimental murine tumour models [22, 23]. Expressed as a 26-kD membrane proform, this proTNF is cleaved by matrix metalloproteinases [24]. The soluble 17-kD TNF cleavage product binds to its main receptors TNFR1 (p55, primary cytotoxic) and TNFR2 (p75, primary pro-inflammatory signalling) [25]. This leads to lipid raft fusion and receptor clustering of the intracellular death domains (Fig. 1), allowing the recruitment of TRADD, TRAF-1/2, RIPK and FADD binding complex I. This complex I dissociates from the TNF receptor and is joined in the cytoplasm by FLIPs and pro-caspase-8 to form complex II, which triggers cell death upon activation of caspase-8. Furthermore, TRAFs connect complex I-signalling to NIK/IKK NF-κB activation (pro-inflammatory, survival) and to ASK/GSK/MKK4&7/JNK signalling (proliferation, survival) [26].

#### Fas and TRAIL as death receptors for apoptotic signalling

Signalling by Fas (CD95/APO1) and TNF receptor share common features. Binding of the Fas ligand (FasL or CD95L) to its receptor CD95 promotes receptor clustering and formation of DISC (core elements: FADD, pro-caspase-8), leading to the reciprocal proteolytic activation of caspase-8. Notably, FADD is recruited directly to the death domain on Fas without the need of TRADD (Fig. 1). TRAIL (TNF-Related Apoptosis Inducing Ligand) receptor signalling is similar to CD95/Fas signalling, except for the existence of decoy receptors that cannot signal to induce cell death. Like in the CD95/Fas system, binding of TRAIL to its receptors DR4 or DR5 induces DISC-formation and rapid apoptosis. The decoy receptors DcR1 and DcR2 compete with DR4 and DR5 for binding to TRAIL, but do not initiate apoptosis [20].

### Caspase family proteases

Cysteinyl-aspartases, known as caspases (proteases that contain cysteine in the active proteolytic centre and are specific for aspartic acid) are central to the execution of the apoptotic process [27, 28]. Caspase activity contributes to most morphological and physiological changes observed during apoptosis at the cellular level, but caspases can also fulfil functions unrelated to cell death [11, 12, 29–31]. Apoptosis may be triggered by extrinsic receptor-dependent or receptor-independent intrinsic pathways. In both cases, the early step in the activation of the caspase cascade is the formation of a multiprotein complex (either DISC or apoptosome) that initiates the reciprocal proteolysis of apical caspases (caspase-8, -9, -10) [13, 32, 33]. Caspase-8 may also participate in the formation of a death receptor-independent cytosolic complex called ‘ripoptosome’, a large ∼2 million Daltons protein complex containing RIP1, FADD, caspase-8/-10 and caspase inhibitor cFLIP isoforms [34, 35]. The ripoptosome will sensitize cells to a variety of death-inducing stimuli [35]. While caspase-8 is essential for death receptor apoptosis, caspase-9 is essential for the mitochondrial signalling pathways. Both pathways join at the site of caspase-3 activation and lead to the activation of other caspases and end-substrates of the apoptotic process [36, 37]. We will provide evidence that demonstrates a role for caspases as modulators of autophagy and programmed necrosis by their ability to proteolytically regulate the activity of signalling molecules.

### Mitochondria and apoptotic signalling

Mitochondria are essential to multicellular organisms for aerobic respiration and disruption of mitochondrial functions leads to cell death [12, 38]. Mitochondria are essential modulators of apoptosis (release of pro-, and anti-apoptotic factors), autophagy (cellular ATP content) and necrosis (mainly due to generation of reactive oxygen species (ROS), but also through the control of cellular ATP level). The mitochondrial apoptotic pathway is modulated by the ratio of pro-apoptotic and anti-apoptotic proteins of the Bcl2 (B-cell lymphoma 2) superfamily. Despite over a decade of research, the chronology of events leading to mitochondrial cytochrome c release is not fully understood. Cytochrome c, together with (d)ATP, bind to the apoptotic protease activating factor-1 (Apaf1) to form a pro-apoptotic complex called apoptosome which specifically activates caspase-9. This initiates a cascade of proteolytic events and results in the activation of the downstream effector caspases-3, -6 and -7 [39, 40]. The apoptosome is also a target for inhibitors of apoptosis proteins (IAPs), which efficiently block the activation and activity of caspase. IAPs can be inhibited by antagonists possessing IAP-binding motifs, such as Smac/DIABLO and Omi/HtrA2 that are also released from mitochondria [41–43]. The intrinsic apoptotic pathway is often activated by the tumour suppressor p53 in response to DNA damage or other cell stressors and can be viewed as a p53-dependent apoptotic pathway.

### Bcl2 proteins

B-cell lymphoma 2 family proteins are important regulators of cell death. Bcl2 was identified at the chromosomal break point (14; 18) (q32; q21) in B-cell lymphomas [44] and is the first proto-oncogene discovered to carry anti-apoptotic functions. So far, more than 20 Bcl2 family proteins have been identified, with Bcl2 being the most widely studied member. Bcl2 proteins can be broadly divided into three groups based on their function and structure: (*i* ) anti-apoptotic proteins such as Bcl2, Bcl-XL, Bcl-W, Bcl-B, Al and Mcl-1 all participate in the prevention of apoptosis by limiting permeabilization of the mitochondrial outer membrane, maintaining the integrity of mitochondria and blocking the release of different apoptosis-activating molecules such as cytochrome c, AIF and Endo G; (*ii* ) pro-apoptotic proteins Bax, Bak and Bok. All Bcl2 family proteins possess at least one (up to four) BH (Bcl2 homology) domains. The anti-apoptotic proteins Bcl2, Bcl-XL and Mcl-1 contain all four conserved BH (1–4) domains, while Bax and Bak possess BH1-3 domains (Table 1); (*iii* ) BH3-only domain containing proteins Bad, Bik, Bid, Bim, Bmf, Noxa, Puma, HRK, Egl-1 and Ced-13 (Table 1).

**Table 1:** Classification of Bcl2 family proteins. Bcl2 family proteins are classified according to their BH domain and their function (see text for details)

BH domains present	Bcl2 family members	Function
BH(1-2-3-4) [238]	BCL2	All Anti-apoptotic
	BCL-X_L_	
	BCL2-L1	
	BCL2-L2 /BCL-W	
	BCL2-L10	
	BCL2-L12	
	BCL2-L13	
BH (2, 4) [239, 240]	BCL-X_ES_	Anti-apoptotic
	BCL-X_AK_	Pro-apoptotic
BH (1,2) [159]	BCL-B	Anti-apoptotic
BH (2,3) [241, 242]	BCL-G_L_	All Pro-apoptotic
	BFK	
BH (1-2-3) [243]	MCL1	Anti-apoptotic
	BAK1	Pro-apoptotic
	BAX	Pro-apoptotic
	BOK/MTD	Pro-apoptotic
	BCL2-A1/BFL-1	Anti-apoptotic
BH (1-2-4) [244]	BCL2-L10/BOO/DIVA	Anti-apoptotic
BH (3) [50]	BCL-X_S_	All Pro-apoptotic
	BAD	
	BID	
	BIM/BOD	
	HRK/DP5	
	BCL2-L11	
	BNIP-1, -2, -3	
	BIK/NBK	
	BLK	
	PMAIP1/Noxa, MAP-1	
	BMF	
	BBC3/Puma	
	NIX	
	Beclin1/Atg6	
	ApoL1	

Critical regulatory roles of Bcl2-family members (especially anti-apoptotic Bcl2 and Bcl-XL) have been exploited in the development of anticancer therapeutics. While numerous inhibitors of anti-apoptotic Bcl2-family members are in various stages of (pre)clinical testing, we would like to mention as examples Genasense (antisense oligonucleotide derivate targeting Bcl2-mRNA), ABT-737, ABT-199 (both are so-called BH3-mimetics) and Obatoclax (small molecule inhibitor of Bcl2-family members). We refer to http://www.clinicaltrials.gov for updated information about clinical trials involving those experimental anticancer drugs.

#### Localization of Bcl2 family proteins in apoptosis

In healthy cells, the anti-apoptotic Bcl2 protein localizes mostly to the nuclear envelope, endoplasmic reticulum and cytosol [45]. Other anti-apoptotic proteins such as Bcl-XL and Bcl-W are localized to the mitochondria and cytosol while Mcl-1 is found in mitochondria, cytosol and endoplasmic reticulum (ER) [46]. Induction of apoptosis initiates intracellular relocation of Bcl2 members. In apoptotic cells, Bcl-XL associates with mitochondria and ER, whereas Bcl-W and Mcl-1 localizes to mitochondria [46]. Upon induction of apoptosis, cytosolic pro-apoptotic Bax protein translocates to mitochondria to insert into the outer mitochondrial membrane to trigger the release of cytochrome c and apoptotic cell death. Detected in mitochondria and the cytosol of healthy cells, the pro-apoptotic protein Bok is re-located mainly to mitochondria in apoptotic cells where it participates in the induction of apoptosis [46]. The apoptosis-promoting Bak protein is localized at the outer membrane of mitochondria and in the ER of healthy cells where it binds tightly to some anti-apoptotic proteins such as Mcl-1 and Bcl-XL, but not to others such as Bcl2, Bcl-W and A1 [47]. Upon induction of apoptosis, Bak is released from its interaction with Mcl-1 and Bcl-XL, enabling the degradation of unbound Mcl-1 and Bcl-XL and allowing free Bak to play a vital role in promoting apoptosis [47]. Bid and Bim are BH3-only domain containing proteins. In healthy cells, Bid localizes to the cytoplasm, whereas, in apoptotic cells, it distributes to both the cytosol and mitochondria. Bid cleavage by caspase-8 in the cytoplasm results in truncated Bid (tBid) which translocates to mitochondria to potentiate death receptor-induced apoptosis through the mitochondrial pathway [48]. Bim is sequestered in the microtubule-associated dynein motor complex [49]. Apoptotic signals induce the dissociation of Bim from this complex and enable Bim to interact with and inactivate Bcl2, thereby enhancing apoptosis [49]. Other BH3-only proteins such as Bik and Hrk localize to the membranes of mitochondria and the ER and their intracellular relocation also contributes to apoptosis.

The molecular mechanisms by which Bcl2-family members regulate apoptosis and other forms of cell death are not universally understood [50, 51]. While agreement exists that the intrinsic apoptotic pathway is modulated by the ratio of pro- and anti-apoptotic proteins of the Bcl2 superfamily, the chronology of events leading to mitochondrial cytochrome c release remains unclear. According to one model, anti-apoptotic Bcl2 proteins (Bcl2, Bcl-XL) bind to and inhibit pro-apoptotic, pore-forming, multi-domain Bcl2 family members (Bax, Bak). Pro-apoptotic BH3-only proteins (Bad, Bid, Noxa, PUMA) compete for the interaction with Bcl2 or Bcl-XL, which results in the release of Bax or Bak. These ‘freed’ pro-apoptotic Bcl2 family members oligomerize at the outer mitochondrial membrane and cause cytochrome c release. Alternatively, the BH3-only pro-apoptotic members interact with and activate Bax and Bak with subsequent mitochondrial membrane permeabilization and cytochrome c release.

### Removal of apoptotic cells

The complete apoptosis process remains an orderly, well-organized process with apoptotic cells being removed by professional phagocytes (tissue macrophages) and neighbouring cells. Molecular signals active in the apoptotic cells such as phosphatidylserine and calreticulin (so called ‘eat me’ signals) aid their recognition by phagocytes [52, 53]. While phagocytes are guided to apoptotic cells by specific ‘find me’ signals (*i.e*. lysophosphatidylcholine) [54], healthy cells are protected by ‘don't eat me’ signals, such as CD47 [55]. However, CD47 may actually participate in the interaction between apoptotic cells and phagocytes [56]. Overall, the molecular basis of the protective ‘don't eat me signals’ remains poorly defined.

## Autophagy

Autophagy (Greek for ‘self-eating’) is an evolutionarily conserved catabolic process of lysosomal degradation of cytoplasmic content in eukaryotes. It is typically a protective, pro-survival response at the beginning; however, if hyperactivated, it will ultimately kill the cell. It is a cellular response to a variety of internal and external stress stimuli like nutrient deficiency, hypoxia, ER stress, oxidative stress [57]. Autophagy is critical during starvation as self-digestion of non-essential and/or unwanted cellular components provides essential nutrients during this period of stress. Autophagy also serves to remove damaged and dysfunctional organelles, misfolded proteins and foreign particles, including microorganisms, thus protects cells against infections [57, 58]. Autophagy plays critical roles in tissue development, differentiation and homoeostasis [59] and modulates health and longevity through ‘housekeeping’ and quality control functions affecting the regulation of innate and adaptive immunity, neuro-degeneration, ageing and cell death [57]. Similar to apoptosis, malfunction of autophagy may contribute to diseases such as ischaemia, cardiovascular diseases, neuro-degeneration and cancer [60, 61]. Depending on the route of delivery of the cytoplasmic material to the lysosomes, several types of autophagy [macroautophagy, microautophagy and chaperone-mediated autophagy (CMA)] can be distinguished [60]. Macroautophagy, which will be discussed here, plays a major physiological role and is better characterized than microautophagy and CMA.

### Molecular mechanisms involved in autophagy

**Fig. 2:**
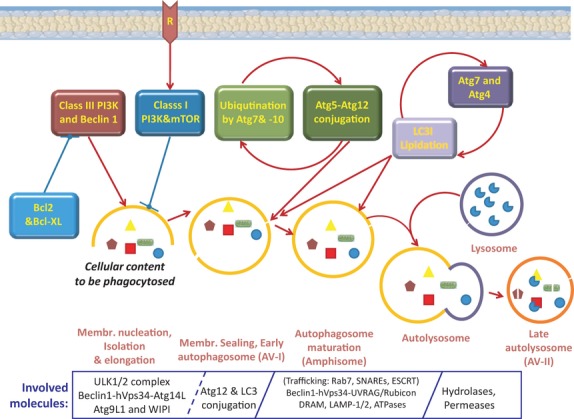
Schematic pathway of different steps of autophagy. Autophagy is initiated by the nucleation of an isolation membrane or phagophore. Autophagy is subdivided into induction, elongation and maturation steps and the role of Atg proteins and Bcl2 anti-apoptotic proteins is shown in each step. Beclin1 and its interrelating associate, class III PI3-K, are required for autophagy induction. Bcl2 anti-apoptotic proteins (Bcl2/Bcl-XL) can attach to Beclin1 and inhibit autophagy. Class I PI3K can negatively control autophagy through mTOR. The initiated isolation membrane (phagophore) then elongates and closes on itself to form an autophagosome. During starvation, this process is initiated by the ULK1/2 complex, the activity of which is controlled by the mTORC1 complex. The Beclin1/hVps34/Atg14L complex, Atg9L1 and WIPI proteins (human orthologs of the yeast Atg18) contribute to the nucleation of the phagophore. Atg12 pathway and LC3 (Atg8) lipidation control the elongation and shape of autophagosomes. Atg12 is essential for the formation of autophagosomal precursors and membrane isolation. Atg7 activates Atg12; activated Atg12 is transferred to Atg10 and conjugated to Atg5 and, finally, a complex is formed with Atg16. The Atg12–Atg5–Atg16 complex is required for recruitment of LC3-II. After autophagosome formation, Atg4 cleaves LC3-II to generate LC3-I. Atg7 activates LC3-I and transfers it to Atg3. The remaining membrane-attached LC3-II is degraded by lysosomal proteases. Rab7, SNARES and ESCRT are involved in the intracellular trafficking along the endocytic pathway. LAMP-1/2 and ATPases are membrane constituents of the endolysosomal compartment. DRAM protein is a lysosomal membrane protein. The Beclin1-hVps34-UVRAG complex positively regulates the maturation of autophagosomes. When associated with the protein Rubicon, it down-regulates autophagosome maturation.

Autophagosomes or autophagic vacuoles (AVs) are double- or multi-membrane vesicles formed during autophagy. AVs sequester components of the cytoplasm and deliver them to the lysosomes for degradation. The formation of autophagosomes involves the following steps: (*i*) initiation, (*ii*) nucleation, (*iii*) elongation and (*iv*) sealing (Fig. 2). The initial step in autophagy is the formation of a phagophore (also called isolation membrane) the origin of which is uncertain [59, 61]. The phagophore expands and encloses the material to be degraded, forming a double-membrane autophagosome also known as ‘early autophagic vacuole’ (AV-I). Dynein motors aid the movement of autophagosomes to fuse with lysosomes to form autolysosomes in which the luminal content is degraded by lysosomal acidic hydrolases. Autolysosomes containing partially digested material are termed ‘late autophagic vacuoles’ (AV-II). The metabolites generated by these degradation processes are released to the cytosol to re-enter metabolic reactions [61].

Although autophagy is known for over 40 years, the precise molecular mechanisms of autophagy-signalling in mammalian cells have started to emerge only within the last decade. The discovery in yeast of a set of AuTophaGy (Atg) -related genes, which were identified as main players in autophagy, has accelerated autophagy research. Currently, most studies on autophagy regulatory molecules have been performed in yeast and have resulted in the identification of 32 different Atg proteins [62–64]. Their roles involve protein turnover, peroxisome degradation, cytoplasm to vacuole targeting (Cvt pathway) and the regulation of size of vesicles/vacuoles and/or peroxisomes [65]. Different Atg proteins have diverse stage-specific functions during autophagy [61]. The ULK-mAtg13-FIP200 complex is required for autophagosome initiation. ULK, mATG13 and FIP200 are mammalian orthologs of yeast Atg1, Atg13 and Atg17 [66, 67]. Atg13 serves to stabilize the complex with ULK1 and FIP200 and phosphorylation of FIP200 and Atg13 by the Ser/Thr kinase ULK is required for the activation of the complex [66]. Another Ser/Thr kinase, mammalian Target of Rapamycin (mTOR), is a well-known cellular energy/nutrient sensor and its activity is directly proportional to the amount of available nutrients. Under high nutrient conditions, mTOR phosphorylates and inhibits Atg13, which prevents its interaction with ULK. AMP activated protein kinase (AMPK) activates ULK through phosphorylation at Ser 317 and Ser 777. The phosphorylation of ULK at Ser 757 by mTOR disrupts this interaction between AMPK and ULK [66, 67]. Starvation or exposure to rapamycin inhibits the activity of mTOR. With the removal of inhibitory phosphorylation signals at Atg13 and ULK, active ULK and Atg13 can interact with FIP200 and the active ULK-Atg13-FIP200 complex now leads to the initiation of the autophagosome formation.

The next steps of vesicle nucleation and assembly are regulated by a complex of Beclin1-phosphatidylinositol-3 kinase (PI3K) and Atg14 [59]. In the absence of a stress stimulus, anti-apoptotic Bcl2 will inhibit the activity of the Beclin1-hVps34/PI3K-Atg14 complex. However, activation of Beclin1-hVps34/PI3K-Atg14 complex occurs under stress conditions when JNK1 causes the phosphorylation and inhibition of Bcl2. The PI3K in this complex produces phosphatidylinositol 3-phosphates (PtdIns3P) and these signalling molecules assist in the recruitment of WIPI-1 and Atg2 to the autophagosomal membrane. WIPI-1 and Atg2 have been suggested to facilitate the recruitment of other proteins and control levels of PtdIns3P [8], albeit their precise roles remain unclear.

The expansion and closure of the autophagosome depend on the two ubiquitin-like conjugation systems Atg12 and Atg8/LC3 (also called: Aut7, Apg8). LC3, unlike Atg12, is present in the early isolation membranes, autophagosomes and autophagic bodies [68], identifying LC3 as a suitable marker for studies on membrane dynamics during autophagy. C-terminal 22 residues of the newly synthesized Atg8/LC3 are cleaved off immediately by autophagin/Atg4 and this generates the active LC3-I cytosolic form [69]. LC3-I is targeted to the autophagosomal membranes in an Atg5-dependent manner and remains there even after the dissociation of the Atg12-Atg5 complex. LC3-I/Atg8 is activated by Atg7 (E1) in an ATP-dependent manner and transferred to the conjugating E2 enzyme Atg3 [70]. Atg7 also activates the other ubiquitin-like protein Atg12 and assigns both, LC3-I/Atg8 and Atg12, to their proper E2 enzymes Atg3 and Atg10 respectively. Final steps involve the interaction of LC3-I/Atg8 with phosphatidyl ethanolamine (PE) [70] for a lipidation reaction, which leads to a conformational change to generate LC3-II/Atg8 (16 kD) [71]. The cycle of conjugation and deconjugation is important for the normal progression of autophagy. So far, Atg8/LC3-II appears to be the only consistent marker of the autophagosome in mammalian cells [72]. The relative amount of membrane-bound LC3-II reflects the abundance of autophagosomes. Both, induction and inhibition of autophagy, may be monitored by measuring total and free LC3-II levels in immunoassays [73]. LC3-II-decorated autophagic membranes fuse with lysosomes and release their content into the autophagosomal lumen [8, 74]. Prior to lysosomal fusing, the autophagosome undergoes a maturation phase by merging with an endosome. This so-called amphisome (Fig. 2) matures to become an autolysosome before it fuses with the lysosome. The regulation of the maturation process of the autophagosome is multi-factorial and involves Rab GTPase, SNARE and ESCRT proteins, molecules of the acidic lysosomal compartment (*e.g*. v-ATPase, LAMP proteins, lysosomal carriers and hydrolases) and Beclin1. The latter modulates the maturation of the autophagosomes by interacting with Rubicon and UVRAG [59, 74].

### Regulation of autophagy

**Fig. 3:**
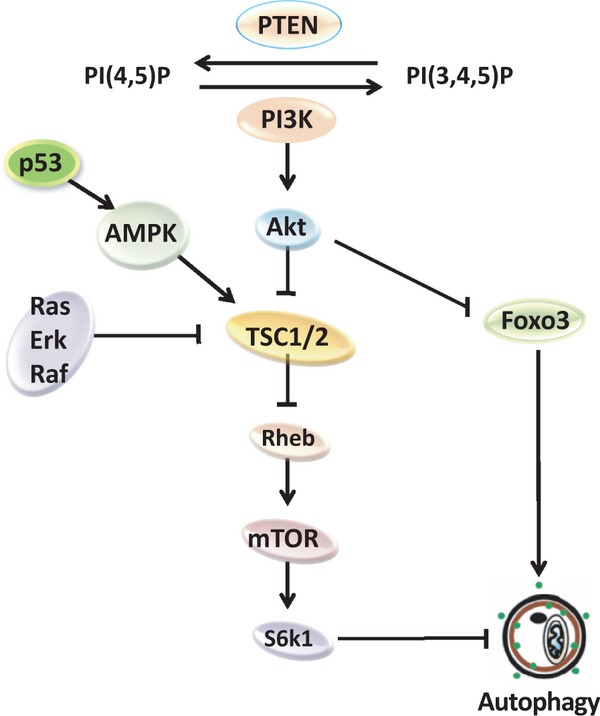
Regulation of autophagy. Autophagy regulation is strongly connected to signalling pathways that promote both cell proliferation (*i.e*. Ras, PI3-K/Akt) and cellular metabolism (*i.e*. S6K; please see the text for details).

Autophagy is regulated by proteins known to play important roles in nutrient signalling, like mTOR, PI3K, GTPases, calcium and elements of protein synthesis machinery [75] (Fig. 3). A sensor of environmental and cellular nutritional and energy status [76], mTOR is a critical metabolic regulator of autophagy [77] and inhibitor of ULK-mAtg13-FIP200 complex assembly and initiation of autophagosome formation. The TOR inhibitor rapamycin and its derivatives activate autophagy and are undergoing clinical trials as anticancer drugs [78, 79]. mTOR can act *via* activation of the p70 S6-kinase (S6K). Under starvation conditions, mTOR activity is down-regulated, but S6K still remains active for some time to ensure that maximal autophagy stimulation is achieved. However, negative cellular feedback mechanisms that inhibit S6K prevent excessive autophagy [75].

Regulatory factors controlling mTOR activity impact on the activation level of autophagy. An important regulatory mechanism of mTOR activity involves the GTP/GDP regulated protein Ras homologue, enriched in brain (Rheb). While GDP-Rheb is inactive, GTP-Rheb potently activates mTOR [80, 81]. Tuberous Sclerosis Complex 1 (TSC1)-TSC2 heterodimer is a negative regulator of mTOR signalling. TSC2 acts as a GTPase-activating protein (GAP) for Rheb converting GTP-Rheb to GDP-Rheb, thus, inhibiting its activity [82–84]. TSC complex is the key coordinator of mTOR signal transduction as various autophagy-signalling pathways are connected through TSC2 (Fig. 3). The Ras/Raf/Erk pathway positively regulates mTOR signalling *via* RSK-mediated phosphorylation of TSC2, leading to the inactivation of the TSC1-TSC2 complex. Erk may also phosphorylate TSC2 and suppress TSC2 function by disturbing the TSC1-TSC2 heterodimer (Fig. 3) [85]. The PI3K pathway positively regulates mTOR signalling *via* Akt-mediated phosphorylation and inhibition of TSC2 (Fig. 3). PTEN, a tumour suppressor and critical regulator of the PI3K pathway [86, 87], selectively hydrolyzes PIP3 to PIP2 and inhibits the activation of Akt/PKB. Akt inhibition leads to suppression of mTOR signalling and the induction of autophagy (Fig. 3). Thus, by down-regulating PI3K/Akt signalling, PTEN has a stimulatory effect on autophagy [88, 89]. Recent studies promote the concept that a phosphatase, possibly PTEN, is inhibited by Bax/Bak. In turn, the resulting up-regulation of the PI3K/Akt/mTOR signalling cascade will cause reduced autophagy [90]. Unlike the Ras/Raf/Erk and PI3K pathways, AMPK pathway has a negative effect on mTOR signalling and promotes autophagy. Upon starvation and activation of calcium signalling, AMPK phosphorylates and activates TSC2 which will inhibit mTOR signalling [91]. The transcription factor FOXO3 has a positive effect on the induction of autophagy. FOXO3 is degraded in cells displaying a hyperactive Akt pathway. In contrast, up-regulation of FOXO3 results in the induction of autophagy-related genes. Intriguingly, the activity of FOXO3 is not influenced by rapamycin suggesting that the autophagy-inducing effect of FOXO3 appears to be independent of mTOR signalling [60].

### Autophagy as a survival response to stress

Depending on various conditions, induction of autophagy may lead to cell death or cell survival. Most studies portrait autophagy as a pro-survival mechanism during stress. Nutrient deprivation generally leads to ROS accumulation and ATP depletion and oxidative stress-induced cell death. Autophagy can prevent cells from undergoing apoptosis by maintaining an intracellular supply of substrates despite the lack of nutrients [92] or blockage of nutrient uptake due to lack of growth factors [93]. Autophagy also promotes the survival of tumour cells under nutrient-deprived conditions. When autophagy (macroautophagy) is inhibited, CMA may still protect cells against some death-inducing stimuli such as ROS and UV light [94]. Autophagy integrates with oxidative stress responses to promote survival of cells during anoikis (detachment of cells from extracellular matrix) [95, 96]. The oncoprotein MUC1 inhibits ROS accumulation and ATP depletion in tumour cells under glucose-deprived conditions and promotes cancer cell survival [97]. These effects of MUC1 are abolished in the presence of an autophagy inhibitor (3-methyladenine) suggesting that during glucose-deprived conditions MUC1 acts *via* autophagy to promote cancer cell survival [97]. The fact that cancer cells utilize autophagy for survival during metabolic stress suggests the potential benefit of autophagy inhibitor strategies for cancer therapy.

## Cell fate and the interplay between autophagy, apoptosis and necrosis

Apoptosis-deficient tumour cells may still die by a process called Caspase-Independent Cell Death (CICD), which is, at least in part, fuelled by ROS generated by damaged mitochondria. Autophagy of damaged mitochondria can counteract the induction of CICD and this is most pronounced in the presence of caspase inhibitors preventing apoptosis [98]. Poly(ADP-Ribose)Polymerase-1 (PARP-1) plays a dual role in modulating UV damage- and oxidative stress-induced cell death. Hyperactivation of PARP-1 leads to ATP depletion and necrotic cell death [99]. However, PARP-1 activation also promotes autophagy *via* the LKB1-AMPK-mTOR pathway to enhance cell survival [100]. The cellular decision of life or death depends on the balance between the autophagy and necrosis mediated signalling pathways. Autophagy may be a last resort for cells to survive stressful stimuli. Selective autophagy towards organelles (especially mitochondria and the ER) has been recognized as an important mechanism in determining cell fate [101, 102]. In hepatocytes, ‘mitophagy’ (autophagy of mitochondria) acts as pro-survival mechanism by limiting the amount of damage incurred by opening of mitochondrial permeability transition pores [103]. Cytotoxic stress (nutrient deprivation, actinomycin D, staurosporin) can produce significant mitochondrial damage and trigger mitophagy in mammalian cells [104]. Thermal cellular damage activates the transcription factor NFκB, which activates autophagy and improves cell survival [105]. Cells lacking NFκB fail to undergo autophagy after heat shock and show increased cell death. In conclusion, basal autophagy acts as a protective mechanism, whereas autophagy deficiency or excess autophagy induced cytotoxic effects.

## Differential effects of autophagy on apoptosis

We will present examples for the currently known three types of interconnections that concurrently determine cell fate based on specific stimuli, environmental cues and cell type: (*i* ) autophagy as an antagonist of apoptosis, (*ii* ) autophagy as a facilitator of apoptosis and (*iii* ) autophagy and apoptosis cooperating to induce cell death (Table 2).

**Table 2:** Interrelations between necrosis, autophagy and apoptosis

Autophagy facilitates apoptosis	Autophagy antagonizes apoptosis & necrosis	Autophagy cooperates with apoptosis
- Maintenance of ATP levels & ‘Eat me’ signal (Phosphatidylserine exposure)	- Energy and nutrient supply (esp. ATP)	- Although separate processes, they may progress in parallel (but apoptosis progresses at faster pace)
- Elements of autophagic machinery are involved in membrane blebbing	- Removal of harmful protein aggregates	- Autophagy upstream of apoptosis: regulation of caspase activity in some systems
- Lysophosphatidylcholine secretion: dead cell clearance/ engulfment	- Prevention of anoikis	- Autophagic cell death is only evident in the absence of apoptosis
- Hyperactivated autophagy may initiate apoptosis		

### Autophagy as an antagonist of apoptosis

Several cellular conditions have identified autophagy as a pro-survival mechanism that antagonizes apoptosis. ER stress induced by environmental cues and chemicals leads to abnormal ER functioning and the formation of misfolded protein aggregates [106, 107]. Removal of misfolded protein aggregates by autophagy can attenuate ER stress, maintain normal ER functions and limit the induction of apoptosis [108–111].

### Autophagy as a facilitator of apoptosis

Autophagy can facilitate apoptosis by maintaining ATP levels during starvation to promote ATP-dependent apoptotic processes. Exposure of the ‘eat me’ signal phosphatidylserine at the outer plasma membrane requires ATP and the inhibition of autophagy results in reduced secretion of lysophosphatidylcholine and impaired clearance of dead cells during programmed cell death in embryoid bodies [112]. Furthermore, Atg5-deficient mice show impaired engulfment of apoptotic bodies during embryonic development [112]. Autophagy might also facilitate apoptosis by enabling membrane blebbing, an ATP-dependent process involving acto-myosin contraction [113].

### Autophagy and apoptosis cooperating to induce cell death

Components of the extrinsic apoptosis pathway, TRAIL, TNF and FADD simultaneously induce autophagy [114–120]. Autophagy and apoptosis may drive cell death and cooperate during this process by acting in parallel or in sequence. Apoptotic and autophagic mechanisms are both induced and required for cell death and full tumour remission in arsenic trioxide-treated T-lymphocytic leukaemia [121], in imatinib treatment of Kaposi's sarcoma [122], and in the effects of vitamin K on leukaemia cells [123]. In CD4^+^ T cells, autophagy is essential for caspase-dependent cell death and appears to act upstream of apoptosis, as cell death with autophagic features is observed upon inhibition of apoptosis. Furthermore, blockage of autophagy by pharmacological inhibitors or specific siRNA knockdown of Beclin1/Atg6 and Atg7 genes profoundly inhibits apoptosis in these cells [124]. Thus, involvement of autophagic cell death may only become apparent under conditions where apoptosis is inhibited, *e.g*. by inhibiting caspase-8 activity [125, 126].

Beclin1 (Atg6) is constitutively inhibited through interactions with Bcl2 or Bcl-XL. Phosphorylation of Beclin1 by DAP-kinase [127], phosphorylation of Bcl2 by the JNK pathway [128] or the activation by Bif-1 [129] weaken these interactions and activate Beclin1. Importantly, the mechanisms relevant to the activation of Beclin1 seem to take place specifically at the ER and do not influence Bcl2 interactions at the mitochondrial membrane [130–135]. Beclin1 may contribute to cell survival or cell death [92, 136–138]. The up-regulation of Beclin1 by the hepatitis B virus protein X can sensitize cells to autophagy and allow them to grow under nutrient-deprived conditions [8, 139]. In contrast, when coupled with Atg5 and caspase-3 inhibition, the lack of Beclin1 can attenuate ER stress-mediated cell death [140] and increase survival of immature neurons [141]. This seemingly dual function in determining cell fate may be explained by the recent finding that Beclin1 can be cleaved by activated caspases at residue Asp^149^, which depletes Beclin 1 of its autophagic function. However, the C-terminal Beclin1 fragment gains the ability to amplify mitochondrion-dependent apoptosis despite the fact that it does not contain a BH3 domain [142, 143]. Upon cleavage, the C-terminal fragment changes cellular localization and no longer interacts with Vps34 in the PI3K-complex. Interestingly, a similar cleavage by caspase-3 was shown to occur in Atg4D, which is thought to activate autophagy. Cleaved Atg4D is highly toxic and this is not a result of enhanced autophagy, but likely caused by its putative BH3 domain inducing apoptosis [144].

BNIP3, also known as Nip3 or Bcl2/E1B-19K-interacting protein 3, localizes to the outer membrane of the mitochondria during cell stress. BNIP3 is a BH3-only member of the Bcl2-family and inhibited by interactions with Bcl2 and Bcl-XL [145, 146] (Table 1) during the induction of autophagy [147, 148] and apoptosis [149–152]. BNIP3 may also induce mitophagy [153, 154] and necrosis [155, 156]. BNIP3 is considered a weaker inducer of cell death than other BH3-only proteins, despite the fact that its BH3 domain appears functional [157, 158]. A tryptophan to aspartate or glutamate (W > D/E) amino acid substitution within the BH3 domain may weaken BNIP3's cell death activity by increasing its affinity for pro-survival Bcl2 family proteins [159]. Phosphorylation of the BH3 domain by survival kinases may also contribute to the attenuation of BNIP3's potency to induce cell death [160, 161]. BNIP3 is unique in that it contains a transmembrane domain which allows it to insert into the mitochondrial outer membrane [162] and homo-/heterodimerize through lateral interactions of alpha-helices [163–166] which collectively contributes to its pro-death activity [163, 166, 167]. BNIP3 expression is most commonly detected during hypoxic cell stress and its expression is directly induced by HIF1-α [149, 168, 169], although HIF1-α-independent pathways have also been described. BNIP3 activation occurs *via* PLAGL2 [170, 171], S100A8/A9 [9, 172, 173], TNF-alpha [147], inhibition of the Akt/FOXO3a pathway [174], nitric oxide [147, 175] and AMPK-mTOR and unfolded protein response pathways [156]. Moreover, repression of BNIP3 activation occurs *via* SIM2s [176], cyclic hyperoxia [177], pRb/E2F [151], the NF-κB pathway [178] and indirectly *via* COMMD1 [179]. In the presence of microtubule inhibitors, BNIP3 can be phosphorylated by a mitotic kinase, which occurs simultaneously with Bcl2 and Bcl-XL phosphorylation. This leads to an increase in BNIP3 stability and increased interaction with phosphorylated Bcl2 [180].

BNIP3 can promote cell death in the myocardium of patients with heart failure [181–183], in neurons [184, 185] and emphysematous lung lesions [171]. Up-regulation of mitochondrial BNIP3 precedes translocation of pro-apoptotic endonuclease G in neuronal death [186]. The repression of BNIP3 promotes a protective form of autophagy [187] that is necessary for basal cell survival [178] as shown for germ cells during spermatogenesis [188]. BNIP3 itself can promote cell survival through the induction of autophagy [164, 169, 189]. This may occur through competition with Beclin1 for Bcl2/Bcl-XL. When targeted to the ER, BNIP3 mobilizes Ca^+2^ from the ER to the mitochondria [185]. This, ER-derived Ca^+2^ release may also be induced by active Bax [190]. Notably, BNIP3 fails to induce mitochondrial dysfunction and cell death in the absence of Bax and Bak [191] or when interacting with Bcl-XL in the presence of the autophagy inhibitor 3-MA, but not the apoptosis inhibitor [N-CBZ-VAD(O-Me) fluoromethyl ketone]. This suggests that crosstalk between Bax/Bak-dependent apoptotic and autophagic pathways may be important for BNIP3 activity.

## Mitoptosis

Mitoptosis (mitochondrial death program) or apoptotic-like changes inside mitochondria is a poorly understood process and currently described based on morphological changes. Induction of mitoptosis and concomitant disruption of ATP supply by mitochondria often induces autophagy to assure maintenance of energy supply [192, 193]. Mitoptosis may occur as an inner membrane mitoptosis with exclusive degradation of the internal matrix and cristae and intact external mitochondrial envelope. In contrast, outer membrane mitoptosis is characterized by swollen internal cristae remnants. The fate of the degraded mitochondria involves either autophagosomal degradation (the predominant phenomenon observed in our lab) or the extrusion of mitoptotic bodies from the cell [194].

## Necrosis

When a cell is unable to die by apoptosis (*i.e*. low ATP-level), it may undergo necrotic cell death [99, 195]. Necrosis can be best defined through its morphology. It is characterized by cell swelling, disfunction (and often swelling and rupture) of mitochondria, rupture of cellular membrane and spill of cytoplasmic content to the extracellular space and cell lysis. Unlike apoptosis, necrotic nuclei do not exhibit condensation, fragmentation and internucleosomal DNA cleavage (no DNA-ladder could be detected), although some DNA degradation occurs in late stages of necrosis. Contrary to apoptosis, necrosis does not involve caspase activation [196, 197]. Inflammatory reactions are frequently triggered in response to necrosis [99, 198]. The induction of necrosis usually takes place in accidental (chemical or physical injury) or acute pathological situations of cell damage [199–201]. Necrosis was long considered an uncontrolled, non-programmed form of cell death resulting in dramatic irreversible alterations in essential cell parameters of metabolism and cell structure [195]. Surprisingly, necrotic cell death can be controlled and molecular events controlling the necrosis program have been discovered [202]. Terms like ‘programmed necrosis’ or ‘necroptosis’ collectively refer to necrosis and emphasize a degree of regulation and molecular mechanism of this death process. Programmed necrotic cell death is the result of the interplay between several signalling cascades with RIPK3, calcium and mitochondria being main players. RIPK3 interacts with RIPK1 and binds to several enzymes of the carbohydrate and glutamine metabolism [203]. Calcium controls the activation of polylactic acid (PLA), calpains and nitric oxide (NO), which induce a series of events leading to necrotic cell death. Mitochondria contribute to necrosis by excessive reactive oxygen species (ROS) formation, PTP (mitochondrial permeability transition pore) and ATP depletion due to mitochondrial dysfunction [204].

### Mitochondria and necrosis

An important activator of necrosis, TNF induces mitochondrial ROS formation, the activation of PARP-1 (poly(ADP-ribose)polymerase-1) and this leads to ATP depletion and subsequent necrosis [195]. ROS formation leads to DNA damage and subsequent activation of PARP-1, a nuclear enzyme involved in DNA repair, DNA stability and transcriptional regulation [99, 196]. PARP-1 activation consumes large amounts of NAD^+^ and induces massive ATP consumption [205, 206]. The resulting cellular ATP depletion favours accelerated necrosis and inhibits energy-dependent apoptotic cell death [99, 207].

### The role of RIPK1 and RIPK3 in necrosis

The serine/threonine kinases receptor-interacting protein 1 (RIPK1) and RIPK3 play important roles in inducing necrosis and are regulated by caspases and ubiquitination. RIPK have three distinct domains: an N-terminal kinase domain, an intermediary RHIM-domain and a C-terminal death domain [200, 203]. TNF or TRAIL stimulates the formation of a RIPK1/RIPK3 complex, an initial step in the formation of a necrosome. RIPK3 is essential for TNF-induced necrosis. Activated RIPK3 interacts with enzymes regulating glycolytic flux, glutaminolysis, and initiates mitochondrial ROS formation [200, 203].

The activity of RIPK1 is specifically associated with necrosis and not with apoptosis. Necrostatin-1 (Nec-1) specifically blocks the kinase activity of RIPK1 [208]. Nec-1 inhibits TNF-mediated necrosis in L929 cells and FasL-induced necrosis in Jurkat cells that were pre-treated with caspase inhibitor zVADfmk or deficient in FADD [209]. RIPK is essential for the TNF-mediated activation of nuclear factor κB (NF-κB). Overexpression of RIPK leads to cell death and RIPK-deficient cell lines are resistant to caspase-independent cell death [203]. The exact RIPK1-activation mechanism remains unknown, but may involve PARP-1 induced metabolic changes resulting in ATP depletion and lower intracellular pH due to lactic acid accumulation under ischaemic conditions which trigger necrotic cell death [202]. Alternatively, RIPK1 activation may occur as a result of enhanced metabolism, *e.g*. activation of glycolysis by autocrine TNF production as a response to cellular stress [210]. This mechanism has been documented in cellular stress induced by caspase inhibitor zVADfmk and ultimately resulted in TNF-mediated necrosis [211].

RIPK1 activity is essential for necrosome formation and homotypic RHIM associations between RIPK1 and RIPK3 are important for stabilizing the necrosome [202]. Under necrotic conditions, RIPK3 also binds to other metabolic enzymes, *e.g*. the cytosolic glycogen phosphorylase (PYGL), the cytosolic glutamate-ammonia ligase (GLUL) and the glutaminolysis-initiating enzyme GLUD1, which positively regulates RIPK3 enzymatic activity [202, 212]. These interactions lead to glutamine production and regulate glycogenolysis. Both, RIPK1 and RIPK3 seem to be responsible for an increased cellular carbohydrate and glutamine metabolism, leading to higher ROS formation and subsequent necrotic cell death [88, 202]. Activity of caspase-8 blocks the necrotic cell death, likely by cleavage of RIPK1 and RIPK3 [200], and downstream, through caspase-3 and -7 activation and PARP-1 [99]. Again, this emphasizes the importance of RIPK1/3 and the enhanced ROS formation during inhibition of caspases for the induction of necrosis [31, 99, 204].

Calcium release from the ER or calcium influx from the extracellular compartment into the cytosol results in the accumulation of calcium in the mitochondrial matrix and causes the opening of permeability transition pores. This increases the permeability of the inner mitochondrial membrane to low molecular mass molecules and offsets the osmotic balance between the matrix and the intermembrane space, leading to swelling and disruption of mitochondria [195]. Increased intracellular calcium concentrations lead to the activation of intracellular, non-lysosomal cysteine proteases of the calpain family, which are ubiquitously and constitutively expressed in mammalian cells [5, 202, 213]. Conserved during evolution, calpains have been shown to contribute to the necrotic cell death of neurons in *C. elegans* [202, 213]. Activated calpains can cleave the anti-apoptotic Bcl-XL and BAX, as well as caspase-7, -8 and -9. It is, however, less obvious if this cleavage inhibits or stimulates caspase activity [202, 214–216]. Calpains also play an important role in ROS-dependent necrotic cell death because they cleave the mitochondrial Na^+^/Ca^2+^ exchanger causing higher Ca^2+^ concentrations in mitochondria and elevated mitochondrial ROS production. Calcium may contribute to necrosis in yet another way: it may stimulate NO synthase activity and enhance NO production. NO is a strong inhibitor of complex IV of the mitochondrial respiratory chain and leads to stronger ROS production at complex III [202].

## Autophagy – a ‘double-edged sword’ in cancer treatment

Some cancer cells undergo apoptosis when autophagic genes are inhibited, whereas others enter apoptotic cell death upon activation of autophagic processes. Autophagy may be induced in cancer cells as an adaptation mechanism leading to resistance against chemo- and radiation therapy. Inhibition of autophagy can sensitize tumour cells to apoptosis and represents a novel way to overcome apoptosis-resistance in certain tumours. Chloroquine and its hydroxylated derivative block autophagy by inhibiting the acidification of lysosomes. The synergistic use of chloroquine and alkylating agents showed a remarkable decrease in tumour growth in mice [217]. The siRNA targeted removal of ATG5 enhanced p53-mediated apoptotic cell death [217]. Drugs such as chloroquine have been used in combination with bortezomib, bevacizumab, paclitaxel, carboplatin and oxalipatin in the treatment of a number of cancers and are currently being tested in clinical trials, either alone or in combination with other chemotherapeutic drugs for treatment of various cancers.

The synthetic glucose analogue 2-deoxyglucose is a potent inducer of autophagy in human glioma and prostate cancer cells [218, 219]. Induction of autophagy by 2-deoxyglucose is mediated by the activation of elongation factor kinase-2 (eEF2-kinase) and the specific siRNA knockdown of eEF2-kinase blocks 2-deoxyglucose–induced autophagy [218]. However, this inhibition of autophagy is followed by a rapid decrease in cellular ATP levels and the enhancement of the cytotoxic effects of 2-deoxyglucose by apoptosis. These results demonstrate that the silencing of eEF2-kinase can shift cancer cells from the prosurvival/autophagic pathway to apoptotic cell death. The combination of metformin and 2-deoxyglucose resulted in p53-dependent apoptosis in prostate cancer cells *via* the energy sensor AMP pathway. 2-deoxyglucose caused autophagy in prostate cancer cells, but metformin inhibited autophagy by the down-regulation of Beclin1 expression and this triggered the shift from prosurvival/autophagy to apoptosis [219]. Consistently, the formation of autophagosomes induces radioresistance [220]. Knockdown of autophagy-related genes such as Beclin1, ATG3, ATG4 and ATG5 will inhibit the formation of autophagosomes [220] and result in the induction of p53-dependent apoptosis upon exposure to radiation. Again, this suggests pro-survival roles of autophagy in some cancer cells. Phenylethynesulfonamide, a small molecule inhibitor of heat hock protein 70 (Hsp70), inhibits autophagy and lysosomal functions and induces cancer cells apoptosis, making it an attractive therapeutic choice for the treatment of cancers with high Hsp70 expression [221].

Many tumour cells display a reduced autophagic capacity when compared with normal cells. Mono-allelic deletion of the Beclin1 gene is found in 40–75% of sporadic human breast and ovarian cancers [222]. Transfection of the Beclin1 gene into MCF-7 cells, which contain low levels of Beclin1, showed that the overexpression of Beclin1 can inhibit tumour growth and tumour formation [222]. These findings confirm the hypothesis that not only have tumour cells lower levels of basal autophagic activity but overexpression of autophagic genes can aid the treatment of certain cancers [222]. Recent studies showed a great variation in Beclin1 levels in colon cancer specimens and Beclin1 transfection was able to inhibit tumour growth [223].

Conventional therapies such as radiation therapy, chemotherapy and targeted therapies are not suitable for tumours displaying profound defects in apoptosis. Especially in these types of tumour cells, induction of autophagy may become an alternative therapeutic strategy. For example, pancreatic cancer is an aggressive malignant disease often resistant to standard chemotherapeutic agents and radiation therapy. The siRNA-mediated targeting of the protein kinase C-δ (PKC-δ) resulted in reduced growth of pancreatic cancer cells by activating autophagy in an apoptosis-independent manner [224]. The Bcl2 proto-oncogene is overexpressed in 50–70% of breast cancers. Overexpression of Bcl2 makes breast cancer cells potentially resistant to radiation therapy, chemotherapy and anti-hormone therapy. The siRNA-mediated Bcl2 silencing potentiated autophagy in the MCF-7 breast cancer cells and knockdown of the autophagy genes ATG5 and Beclin1 inhibited autophagy in Bcl2-depleted cells [225]. Doxorubicin induces autophagy and apoptosis at lower and higher concentrations, respectively. Low dose doxorubicin treatment in combination with Bcl2-knockdown increased autophagy and reduced tumour growth [225]. The von Hippel-Lindau (VHL) tumour suppressor gene is inactivated in 75% of the renal cell carcinomas. The small compound STF-62247 specifically targets the VHL-deficient cancer cells by inhibiting growth and inducing extensive autophagy [226]. VHL-deficient cells exhibited higher acidification of autolysosomes in response to STF 62247 and knockdown of autophagy genes such as ATG5, ATG7 or ATG9 prevented the autophagy induction by STF 62247 in VHL-deficient cells [226].

## Cellular context matters in irradiation-induced cell death in clinical settings

Irradiation kills (cancer) cells mostly by the induction of radicals that cause damage primarily to DNA, but also to other molecules. Like in chemotherapy, the outcome of radiotherapy is dependent on the cellular p53-status. In normal cells, p53 acts to stop the cell cycle, activate the DNA-repair machinery, and, if the damage persists, initiates the expression of apoptotic genes to remove damaged cell. Many types of cancer lack a functional p53 or carry mutations that abolish various functions of p53. This prevents them from committing to rapid apoptosis upon irradiation, but, instead, ignore cell cycle checkpoints and continue through the cell cycle [227, 228]. Unrepaired or miss-repaired DNA damage and lack of functional checkpoints (especially at G2/M and during mitosis) will lead to imbalances in segregated genetic material and mitotic catastrophe. The inability of cells to complete mitosis may (*i*) delay apoptosis, (*ii*) engage necroptosis, especially when the apoptotic machinery is damaged or inhibited and (*iii*) initiate cellular senescence [229]. Adding to this complexity is the fact that epigenetic regulation results in heterogeneous P53 gene expression profiles within the same tumour [230]. To address this complexity, the design of future curative anti-tumour treatment must include patient- and tumour-specific multi-modular therapeutic targeting regimes that address the interconnections of different cellular death pathways aimed at forcing tumour cell death.

## Stress-induced cell senescence

While therapies should ideally kill cancer cells, their induction to undergo permanent/irreversible cell cycle arrest called cellular senescence is also an attractive therapeutic option (what does not proliferate, will not kill the patient). Repeated DNA damage alone, an event frequently triggered by various classes of anticancer drugs, may induce senescence in cancer cells [231]. Apparently, this is achievable at concentrations much lower than those used for classical anticancer therapeutic regime, but still sufficient to trigger persistent replication stress (detected by histone H2AX phosphorylation and induction of p21^WAF1^), but low enough not to suppress protein synthesis. Moreover, senescence accompanying cancer therapy can be detected *in vivo*. For example, senescent cells were seen in over 40% of biopsies from patients with breast or lung cancer that had been treated with cytotoxic chemotherapy [232, 233]. Thus, treatment with high drug concentrations designed to kill cancer cells, may trigger senescence in a subset of cells.

Persistent DNA-damage-induced cellular senescence relies on pathways also involved in autophagy- and apoptosis regulation. It is an effect of both, inhibition of cell cycle progression and the ongoing activity or activation of mTOR-regulated pathways [234–236]. In the absence of mTOR activation, inhibition of cell cycle progression is by itself not sufficient to generate cellular senescence [234]. However, oncogenic pathways hyper-regulated in cancer, also promote mTOR activation (increased protein synthesis, S6-signalling), thus facilitating the induction of senescence [237].

## Clinical relevance of cell death modulation

Much attention has been devoted to experimental drugs modulating autophagy or apoptosis. Several drugs have proven promising during experimental anticancer and anti-degenerative therapies, but unexpected results have also been observed. Here, we propose that some of these unforeseen effects are due to the interconnected nature and molecular regulation of death pathways and the contradictory roles of autophagy in cell survival, which depend on the particular cellular context (*i.e*. pro-survival roles of low-level autophagy *versus* pro-death signals released by extensive autophagy activation). The current data imply that autophagy-modulating anticancer and anti-degenerative drugs will likely be most effective when applied in conjunction with carefully selected other therapeutics to induce synergistic effects.
